# Supplementary material for: Are carbon emissions trading and green financial instruments synergistic? -Comprehensive quantitative research based on content analysis
Source: PLoS One. 2024 Mar 7;19(3):e0298601. doi: 10.1371/journal.pone.0298601 (PMC10919601; doi:10.1371/journal.pone.0298601)
Supplement: S1 Data — (ZIP) [file pone.0298601.s001.zip › Data/地级市Z_F工作报告.zip!/░▓╗╒/║╧╖╩╩╨/└┤╫╘╣1⁄2╓┌║┼í╛┬φ┐╦╩2╛▌═°í┐_2008.docx]

# **2008年政府工作报告**

发布时间：2008-02-01 17:03

【字体：[大](http://www.hefei.gov.cn/public/1741/javascript:void(0);) [中](http://www.hefei.gov.cn/public/1741/javascript:void(0);) [小](http://www.hefei.gov.cn/public/1741/javascript:void(0);)】

各位代表：

　　现在，我代表市人民政府，向大会作政府工作报告，请予审议，并请市政协委员和其他列席人员提出意见。

　　一、过去五年及２００７年工作回顾

　　过去的五年，是改革开放以来我市经济社会发展最好最快的时期。在省委省政府和市委的坚强领导下，我们高举邓小平理论和“三个代表”重要思想伟大旗帜，认真贯彻党的十六大、十七大精神，把握发展第一要务，坚持科学发展，坚持改革开放，落实中央各项方针政策，大力实施“工业立市”、“创新推动”、“县域突破”、“１４１”空间布局等重大发展战略。精心谋划“十一五”发展，启动建设“省会经济圈”，开展“大拆违”，实施“三大推进”，全市人民解放思想，抢抓机遇，开拓创新，十三届人大确定的主要经济指标全部提前完成。

　　——这五年，是经济实力实现大步跨越的五年。与２００２年相比：地区生产总值增长１．２倍；人均生产总值突破３５００美元，增长一倍多；固定资产投资增长６．８倍；财政收入增长２．５倍；税收收入增长２．６倍；全社会工业增加值增长１．４倍；产业结构进一步优化，三次产业比例由８．１∶４３．１∶４８．８，调整为６∶４９∶４５；家电、汽车、工程机械等产业综合产能进入全国前列；高新技术企业发展到４２７家，产值增长近三倍，占规模以上工业总产值比重超过５０％；建筑业产值增长３．５倍；县域生产总值增长１．５倍，增速高于全市２．８个百分点，比重上升３．７个百分点。省会经济首位度超过１８％，提高近四个百分点。

　　——这五年，是改革开放取得突破性进展的五年。政府职能转变加快，行政管理体制改革提速。公共财政体制、投融资体制、城市规划建设体制、招投标体制、土地储备交易制度、城市管理综合执法体制、基础教育招生及事业单位等改革整体推进，发展的“大环境”不断优化。国有、集体中小企业产权改革基本结束，以合钢主业重组为标志的大企业改革强力推进，市属上市公司股权分置改革全面实现，生产经营类事业单位转企改制工作初步完成。农村综合改革成效显著，新农村建设新机制初步形成。对外开放向纵深拓展。与奥斯纳布吕克等三座城市结为友好城市，开通“港澳自由行”，实现与长三角地区一体化通关；三大开发区和七个县区工业园区成为对外开放的排头兵。五年来，全市累计利用外资２７．５５亿美元，引进内资超千亿元，进出口总额２１４亿美元。

　　——这五年，是城乡面貌发生巨大变化的五年。城区规划面积扩大３８０平方公里，建成区面积新增７７平方公里，城镇化水平提高１１．３个百分点。成功开展全国闻名的“大拆违”，拆除各类违法建设１３８０万平方米，实现“零补偿、零冲突、零事故”。城市主干路网新建、扩建全线展开，小街巷改造基本完成，人均道路面积增长２６．１％。主城区改造提升加速推进，政务文化新区基本建成，３．５平方公里滨湖新区启动区英姿初现，四大组团全面拓展，“１４１”城市发展空间布局初露端倪。环城高速、宁西铁路建成通车，一环六射高速路网格局初步形成，区域综合交通枢纽地位逐步确立。生态建设成效明显，森林覆盖率达到１５．５％，城区人均公共绿地面积净增３．６平方米，日污水处理能力新增２３．５万吨。农村基础设施大为改善，农业综合生产能力大幅提高，乡镇及７０％以上行政村修通水泥路，广播电视实现“村村通”，新农村建设示范典型不断涌现。

　　——这五年，是人民生活水平大幅提高的五年。五年来，城镇居民人均可支配收入增长８７．５％，农民人均纯收入超过全国平均水平，增长近一倍；人均储蓄余额增长１．１倍，居民家庭财产普遍增加；社会消费品零售总额增长１．１倍，住房、汽车等高档消费需求迅速升温。综合性连锁经营等新型流通业态和物流、会展、旅游、金融等现代服务业发展加速，较好地满足了多样化消费需求。促进就业的长效机制初步建立，新增城镇就业岗位３５．５万个。社会保障体系进一步健全，“五险”参保人数净增１０５万人次；企业离退休人员养老金、城镇职工最低工资、城镇低保标准分别提高５８％、４７．３％和６４％。城市廉租房累计配租６９２４户１６９１０人次，做到应保尽保。

　　——这五年，是精神文明及和谐社会建设成果丰硕的五年。文明创建取得新的进步，全社会文明程度进一步提高，蝉联“全国创建文明城市工作先进市”称号，荣膺“全国双拥模范城”六连冠。科技创新能力显著增强，成为全国唯一的科技创新型试点市，成功举办一年一度高新技术项目—资本对接会。“文化强市”初见成效，公共文化产品和服务不断丰富。成功获得第四届全国体育大会承办权，建成市体育中心、赖少其艺术馆等一批重大文体设施。教育事业健康发展，布局调整和资源均衡化成效显著；新增中小学校舍１２２万平方米，创建省、市示范高中１８所，特色初中、小学２６所。基本建成城乡医疗救治等三大网络体系，城市社区卫生服务网络覆盖面达到８５％，新型农村合作医疗制度参合率突破９０％。平安合肥建设成效明显，社会持续和谐稳定。荣获全国社会治安综合治理优秀城市，捧回首届“长安杯”。

　　刚刚过去的２００７年，我们继续强力推进“大发展、大建设、大环境”，致力扩大有效投入，着力提升城市综合承载力，全力加大城乡统筹，努力增强发展活力，倾力实施民生工程，大力推进生态环境建设，经济社会发展进一步加快、效益进一步提升、人民收入进一步提高、社会更加稳定和谐，多项指标创近年来最好水平。预计，全年实现地区生产总值突破１３００亿元，较上年增长１７％以上；财政收入２１５．２亿元，增长２８．３％，其中，地方财政收入１０１．９８亿元，增长２８．４％；全社会固定资产投资１３１０亿元，增长５８．８％；社会消费品零售总额４６５亿元，增长２１％；居民消费价格指数１０５．６；城镇居民人均可支配收入１３４００元，增长２１．７％；农民人均纯收入４４００元，增长１９％；城镇登记失业率４．１％，城市综合实力进一步提升。

　　一是“工业立市”成效显著，科技创新取得新进展。促进新型工业化发展的政策体系进一步完善，工业发展速度快、效益好、后劲足。市、县（区）财政均全力支持工业经济发展，其中，免收（代交）２２００个企业投资项目行政事业性收费２．６亿元，是２００６年的１．６６倍。强力实施“项目推进年”活动，全年完成工业投资３４０亿元，增长７６％；其中，“１２１”项目投资７３亿元，是年度计划的１１８％，江汽、格力、美菱、美的等一大批重点项目加速建设。全市工业增加值突破５００亿元，增长２３％以上，其中，规模以上工业实现增加值４６５亿元，约占全省２０％，增长２６％。八大产业及高新技术产业快速发展，高新技术产业产值超过８００亿元，增长３０％。三大开发区和七个县区工业园区完成工业增加值２９７．７亿元，增长２８．３％。规模以上工业经济效益综合指数高于全省平均水平４９．３个百分点。新增规模以上工业企业２３４户、产值超亿元企业２５户。

　　继续加快科技创新型试点市建设。在肥召开了部际协调小组第一次会议；出台了《试点市工作任务分解意见及考核办法》和《科技创新专项基金管理办法》等一系列政策性文件，创新型城市建设的政策支持体系更加完善；创新型企业培育计划初见成效，首批３５户通过验收，全年新增高新技术企业７０家；进一步强化产学研合作机制，新建省级工程（技术）研究中心五家，秸秆生产重油替代品等多项高新技术成果实现产业化；专项安排２５００万元财政资金，启动科技创新公共服务中心建设；一批高水平企业孵化器迅速成长，合肥民营科技创业服务中心被认定为国家级高新技术创业服务中心；科技创新示范区建设步伐加快，合工大汽车工程研究院等六个产学研项目建成运行；“城市一卡通”工程开始实施，现代科技馆开工建设；创建国家知识产权示范城市取得成效，全年实现专利授权７５０件。

　　二是城乡统筹力度进一步加大，新农村建设实现新突破。整合和改进支农方式，加大新农村建设支持力度，全年市财政安排涉农资金４．３９亿元。打破城乡二元结构，推进城乡一体化进程，初步实现规划、产业、基础设施等城乡对接。以城市化理念引领县域城乡规划建设，以城市大工业带动县域产业配套发展，实现了城市供水、供气、污水管网等公用设施与县域及周边工业园区对接，城市公交车直达肥东店埠、肥西上派及长丰双墩镇。

　　县域经济发展继续提速，预计全年实现生产总值２８０亿元，增长２０％。县域规模以上工业增加值突破百亿元，增长３８％，占全市比重提高２．８个百分点，对县域经济增长贡献率超过６０％。农业产业结构进一步优化，粮食总产再创历史新高；瓜菜果设施栽培面积新增１．６万亩，“一村一品”特色种植村发展到１３５个，新建农产品无公害生产基地六个，本地蔬菜市场占有率５０％以上；新增生猪、家禽规模养殖场（区）５６０个、百头以上奶牛养殖小区２０个，养殖业占全市农业总产值５５％以上；苗木交易大会、葡萄节、龙虾节等会展休闲观光农业呈现蓬勃发展势头。完成植树造林１．１８万亩，新建乡村绿色长廊林带２４３公里。农业产业化步伐进一步加快，新增市级以上龙头企业４０家。

　　新农村建设由点到面快速拓展，市级示范点扩大到１０１个，县区示范点发展到３００多个。出台并实施“村庄环境整治行动计划”，在４００多个村庄开展以“四清四改”为主要内容的环境整治，农民居住条件大为改善。农村基础设施建设进一步加强，瓦埠湖综合治理罗塘保庄圩工程进展顺利，实施中小水库除险加固４８座，“村村通水泥路”突破１０００公里。

　　三是各项改革深入推进，对外开放水平进一步提高。大力开展“改革创新年”活动。实施新一轮政府机构改革，调整１６个政府部门职能，政府效能建设进一步深入，依法行政全面加强。投融资体制改革继续深化，“借、用、还”一体化的管理体系进一步向县（区）延伸，东华科技成功上市，科技农村商业银行挂牌运营。招投标市场管理制度进一步健全，政府性建设与采购资金节约率１５％以上。机关事务管理改革扎实推进，后勤运行成本有效降低。设立国有资产管理机构，优化国有资本运营。国有、集体企业改革进一步深化，国风、荣事达等大企业改革取得阶段性成果。市政、园林系统“管养分离”试点稳步实施，公用事业企业主辅分离改革继续深化，企业生活区移交辖区管理工作加快推进。农村集体林权制度改革任务基本完成。文化体制、开发园区管理体制改革开始启动。

　　对外开放和招商引资取得新突破。成功举办家电博览会等一系列国际性会展。推进区域旅游城市协作，引进海航集团等战略伙伴，增开港澳国际航班。组建第四批招商小组３２８个，增设驻外联络处八个，招商引资５２５亿元，实际利用外资１０．１２亿美元，分别增长５７．８％和４０．２％。大陆集团、麦德龙等世界５００强来肥投资发展，日立建机、联合利华等外企再度增资扩股。全年进出口总额５８亿美元，增长１９％，成为“中部地区加工贸易梯度转移承接地”、“国家级汽车及零部件出口基地城市”和“中国服务外包基地城市”。

　　四是“１４１”战略加速实施，滨湖大城市建设扎实推进。完成城市总体规划、近期建设规划和３６个专项规划，编制土地利用总体规划大纲，城市建设做到“两规合一”、依法有序。以道路建设为重点，“大建设”高品位、快速度推进，全年完成新建、续建道路１２５条、桥梁２４座。区域综合交通枢纽建设稳步推进，合六叶高速全线竣工，３１２国道大陇段“４改８”加速施工，合宁高铁基本建成、春节前可望通车，新桥国际机场完成前期工作即将动工建设，合肥港完成规划；蒙城北路、徽州大道、包公大道建成通车，金寨路高架即将实现“双贯通”，“１４１”组团“一刻钟快速交通网”基本形成；城市出入口道路建设全面拉开，合安、合马、合水（双水段）路全线通车，合六路开工建设；主城区路网工程建设进一步加快，黄山路、胜利路等建成通车，中环线实现全贯通，“一环畅通工程”进展顺利。坚持“四个优先”，高起点、大手笔推进滨湖新区规划建设，启动区内１１条道路构成的路网结构形成，滨湖三校按期招生，２８万平方米拆迁恢复楼如期建成，规划面积４４万平方米的廉租房和经济适用房项目开工建设。安纺一、三、四村等老城区危旧小区和城中村改造加快推进，共改造面积５４．７万平方米，受益居民近５０００户。城市公用设施建设继续推进，五水厂基本建成，六水厂及市府广场ＢＲＴ公交枢纽开工建设，义兴、瑶海公交停保场投入使用。结合道路建设，城市供电系统改造稳步推进。整合供热资源，扩大供热范围，供气、环卫设施建设进一步加强。

　　五是环境综合治理力度加大，可持续发展能力进一步增强。围绕现代化滨湖大城市确立的生态宜居目标，切实转变发展方式。制定实施方案，建立工作责任制，深入推进钢铁、电力、化工等重点行业节能减排工作。关闭淘汰落后水泥产能１４万吨、落后钢铁产能４８万吨，关停小火电机组４１．１万千瓦时。３８家耗能大户落实技改项目１３９项，总投资４２亿元。马钢（合肥）公司总排口污水处理回用工程基本建成，使其占全市５１％的工业污水实现零排放。深入开展小化工整治、水源地保护等专项行动，查处了一批环境违法行为。城市集中供热范围内的燃煤锅炉加快淘汰，公交、出租车等行业“油改气”稳步实施。节能５０％的建筑设计标准全面施行，全年新增节能住宅９００多万平方米。以巢湖为主体的水环境治理力度进一步加大，南淝河沿岸截污工程加快建设，新建城市污水管网３２３公里；清溪路垃圾填埋场综合整治工程全面开工；龚响堂污水处理厂、蔡田铺污水处理厂一期建成试运行，望塘污水处理厂二期及五个小型污水处理厂开工建设。单位ＧＤＰ能耗降幅、主要污染物排放量削减幅度均超额完成省里下达任务。

　　土地资源的节约集约利用得到切实加强，城乡统一、公开公平公正的土地市场进一步健全。实行工业用地最低限价制度，全面推行经营性用地推介和“招拍挂”出让制度。细化土地出让合同，强化供后监管，依法清理闲置土地２１７宗１．２万亩。土地开发复垦整理取得新成效，全年新增耕地近四万亩，连续第八年实现“占补平衡”。再生资源回收利用体系试点工作稳步推进，热电厂污泥焚烧项目基本建成，节水型城市建设及公共交通周、“无车日”等活动积极开展。城市园林绿化取得新进展，实施大蜀山森林公园一期、高压走廊二期、翡翠湖、蜀峰湾、政务区环区河道公园等一批重点建设工程，新增园林绿地一万多亩。

　　六是文明创建扎实推进，各项社会事业全面进步。各项群众性创建活动深入开展，“四乱”及市容环境等专项整治成效显著，通过首届全省文明城市检查验收。公民道德教育、诚信建设得到进一步加强，涌现出徐辉等一批道德楷模。学习型城市创建活动继续扎实开展，“庐州讲坛”、“科学家企业家讲坛”成为品牌。文化事业进一步发展，广场文化、节日文化等群众性文化活动精彩纷呈。文化艺术中心、广电中心等设施加快建设，卫星广播电视转星调整工作顺利完成，数字电视整体转换率达到８２％。大力推进文化产业发展，成为“国家动漫产业基地”。教育布局进一步优化，优质资源快速扩张，３２所小区配套中小学全部投入使用。积极关注弱势群体子女教育，农民工子女义务教育定点学校增加到７０所。职教基地建设快速启动，省职业技术学院等１５所院校签约入驻并已有六所相继开工。新一轮卫生基础设施建设全面展开，建成市口腔医院新院，开工市中心医院、职业病防治大楼；支持安医大第二附院、省立儿童医院、安徽心脑血管医院等加快建设。全民健身活动广泛开展，参赛全国六城会获六金四银七铜佳绩。人口与计生工作稳步推进，成为全国婚育新风进万家活动示范市。国防动员、民兵预备役、人防、社会慈善工作进一步加强。妇女儿童、老年人、残疾人权益得到保障，统计、保密、对台、民族、宗教、外事、侨务、防震减灾、气象、档案、地方志、科普等工作都取得新成绩。

　　七是优先实施民生工程，和谐合肥建设稳步推进。出台１５项配套政策，各级财政投入十二项民生工程建设资金５．６亿元。启动实施城镇居民医疗保险制度，全市参保人数超过６７万；建立农村居民最低生活保障制度，受益农民８．１万人；提高城乡散居“五保户”供养标准，集中居住、供养的五保对象全部纳入城市低保范围；完善城镇未参保集体企业退休人员基本生活保障制度，对符合条件的２６７９人按时足额发放基本生活费；推进城乡卫生服务体系建设，新建社区卫生服务机构２８所，改造乡镇卫生院和村卫生室１１４所；扩大城乡医疗救助范围，兑现救助资金１２００万元；开展重大传染病人医疗救治和生活救助，提高生活救助标准；继续消除农村中小学危房，改造Ｄ类危房７．０５万平方米；完善农村部分计划生育家庭奖励扶助政策，市财政专项安排配套奖励资金；实施农村饮水安全工程，１８万人安全饮水问题得到解决；建立义务教育经费保障机制，免除５３万名城乡学生学杂费及６．６万名贫困家庭学生教科书费，对５５１１名贫困寄宿学生给予生活补助。

　　全面落实就业再就业优惠政策，全年城镇新增就业岗位８．５３万个，帮扶困难群体再就业１．５万人。创建退管示范社区１００个，退管服务率９０％以上。加强生猪、蔬菜生产及市场供应，努力缓解副食品市场价格上涨压力，对部分生活困难群体实行发放一次性肉价补贴等措施。出台廉租房和经济适用房实施意见，编制全市解决城市低收入家庭住房困难发展规划和年度计划，继续足额发放符合条件困难家庭住房补助。切实维护农民工合法权益，清理拖欠农民工工资２８００多万元。

　　社会治安防控防范体系进一步健全，全市社会治安秩序持续良好。继续推行领导干部公开接访、带案下访，深入开展矛盾纠纷排查化解工作和信访疑难复杂问题终结息访专项行动，切实维护群众利益，信访秩序进一步好转。公共突发性事件应急管理中心建成并投入使用，成功开展了危化品泄漏应急演练。深入开展产品质量和食品安全专项整治行动，全面完成国家和省确定的整治目标任务。消防基础设施进一步完善，新建消防站八座。重点行业和领域安全生产隐患排查治理力度进一步加大，安全生产形势总体平稳。

　　八是政务环境进一步优化，民主法制建设取得新成绩。广泛开展“查摆找补创”活动，集中整改解决项目落地、窗口服务、工作流程及涉及群众切身利益等方面存在的问题。行政审批制度改革进一步深化，优化并联审批流程，大力推行“柜员制”、“首席代表制”、“一次性告知制”。再造机关对外办事和内部工作流程，促进管理模式向精细化、扁平化、项目化转变。加大政府目标管理和督查力度，严格执行行政责任追究制。强化行政监察职能，规范市直机关公务员津补贴发放，市本级３５２家行政事业单位非税收入全部纳入新的征管系统。加强重点行业、重点部门、重点项目的审计监督，查处了一批违纪违法案件，挽回直接经济损失２４３９万元。清理确认并向社会公布了市本级９１个行政执法主体、４１７４项行政职权目录。开通“阳光政务１１４热线”，被命名为“全国政务公开示范点”。全面清理和规范评比达标表彰，共撤并５９１项。

　　坚持依法行政，自觉接受人大法律监督、政协民主监督、社会舆论及人民群众监督，加强与各民主党派、工商联和无党派人士的沟通联系。全年共办理人大代表议案、建议１５３件、政协委员提案４０６件；民主党派综合大楼建成投入使用。积极支持工会、共青团、妇联等人民团体依据章程创造性开展工作。加强政府立法工作，制定政府规章５件、清理８３件。全面落实“五五”普法教育任务，社区居民自治、村民自治及基层民主政治建设扎实推进。

　　各位代表，回顾过去的五年，成绩来之不易。这些成绩的取得，是省委省政府和市委正确领导的结果，也是全市广大干部群众共同努力的结果。在此，我谨代表市人民政府，向全市广大工人、农民、知识分子、干部、驻肥解放军指战员、武警官兵和政法干警，向各民主党派、工商联、人民团体和社会各界人士，致以崇高的敬意！向所有关心支持合肥改革开放与现代化建设的海内外朋友，表示衷心的感谢！

　　在肯定成绩的同时，我们也清醒认识到，我市发展还面临着很多挑战和问题，主要有：经济总量不够大，综合实力不够强，主导产业优势不够突出，人均产出水平不高；农村基础薄弱，农民收入偏低；城市综合承载力较弱，工业园区等平台建设有待进一步加强；水环境综合治理压力较大；特别是观念、体制、机制等制约发展的深层次问题仍然存在，优化环境的任务依然较重。如果这些矛盾和问题得不到有效解决，快速发展的势头就很难保持下去，甚至可能出现转折。尤其是当前，宏观经济环境总体趋紧，区域发展竞争日趋激烈，制约发展的不确定因素明显增多。我们必须增强忧患意识，进一步解放思想，创新发展思路，迎难而上，勇于开拓，及时化解前进道路上的各种困难和问题，才能实现新的跨越。

　　二、今后五年的指导思想和主要奋斗目标

　　今后五年，是我国全面建设小康社会的关键时期。综观全局，国际资本向发展中国家流动的范围加大，沿海发达地区产业向中西部地区转移的速度加快，国家统筹区域发展进入实质性阶段，中部崛起战略在加速推进，尤其是新一轮宏观调控，为我们后发地区的发展提供了新的契机。这些都为我们进一步发挥已有的区位、交通、科技、产业和环境等优势，谋求更好更快发展提供了广阔的空间。凝心聚力，抢抓机遇，乘势而上，奋力崛起，是全市人民的共同愿望，更是我们义不容辞的责任。今后五年，政府工作总的指导思想是：以党的十七大精神为指引，高举中国特色社会主义伟大旗帜，深入贯彻落实科学发展观，紧紧围绕省第八次党代会、市第九次党代会确定的目标任务，继续坚持既定重大发展战略，继续坚持“三大推进”，进一步解放思想，深化改革，扩大开放，求真务实，加快建设全国重要的先进制造业基地、高新技术产业基地、现代服务业基地和区域综合交通枢纽，积极构建省会经济圈，在安徽崛起中勇当先锋，在中部崛起中争先进位，努力向经济繁荣、人民富裕、功能完善、生态良好、文明开放、社会和谐的现代化滨湖大城市目标迈进。

　　未来五年主要奋斗目标是，到２０１２年，地区生产总值年均增长１６％以上，人均生产总值达到６万元；财政收入年均增长１５％以上；城镇居民人均可支配收入增长１２％以上，农民人均纯收入年均增长１３％；人口年均出生率控制在１２‰以内。与２００７年相比，单位ＧＤＰ能耗降低２０％；主要污染物排放降低１２％。

　　——继续强化“工业立市”战略，基本形成优势产业格局。家电、汽车、装备制造等重点产业集群形成较强竞争力，培育千亿产业一个、５００亿以上产业两至三个；电子信息、新材料、新能源等产业加速发展，高新技术产业增加值占全市工业增加值６０％左右。开发园区发展实现新跨越，三大开发区综合实力继续提升，县区工业园区特色产业集群更具规模。农业多种功能得到开发，农业现代化进程进一步加快，实现城郊型农业向现代都市农业转变。

　　——不断深化改革，基本建立科学发展的新体制、新机制。加快重点领域和关键环节的改革步伐，着力构建充满活力、富有效率、有利于又好又快发展的体制机制。科技创新型试点市建设深入推进，城市创新发展能力显著增强。人才、资本、技术、土地等市场体系进一步健全，要素分配和激励机制更加完善，各类市场主体活力充分释放。创新创业氛围更加浓厚，公平竞争格局进一步形成，非公有制经济占经济总量份额较大提升。行政效能显著提高，发展环境不断优化。

　　——进一步解放思想，形成全方位对外开放新格局。对外交流与合作的区域不断扩大，城市开放度进一步提高。东向发展取得新突破，以构建省会经济圈为起跳点，与长三角地区实现良性互动发展，成为沿海地区产业资本转移的首选地之一。利用外资质量和水平全面提升，开放型经济加速发展；新型流通业态、物流、旅游、会展、金融等产业发展水平进一步提高，基本建成区域性商贸物流中心、旅游会展中心和金融中心。

　　——统筹城乡发展，现代化滨湖大城市建设初见成效。基本建成区域综合交通枢纽和“１４１”城市框架体系。建成新桥国际机场、合肥港综合码头、合肥新铁路枢纽及合蚌高铁，完成一环六射高速公路建设；建成以快速路、一级主干路组成的城市快速通道体系，城市道路交通系统更加完善。“１４１”组团联动发展，中心城区品位明显提升，四大组团发展能力显著增强，滨湖新区拓展区初步建成。市域城镇体系进一步完善，城镇化率达到６５％左右。新农村建设全面推进，继续保持全省领先水平。

　　——坚持可持续发展，生态文明建设取得新进展。基本形成节约能源资源和保护生态环境的产业结构、增长方式和消费模式；循环经济初具规模，土地等资源可持续发展的新机制基本建立。城乡一体森林生态网络初步建成，全市森林覆盖率１８％以上，城市绿化覆盖率４５％以上；以巢湖综合治理为龙头的水环境治理取得重大成效，城市重点污染源废水、废气达标率９５％以上，生活污水集中处理率９０％以上；成为国家环保模范城市和生态园林城市。

　　——高度关注民生，和谐社会建设再上新台阶。积极推动文化大发展、大繁荣，促进城市文化品位不断提升，区域性文化中心初步形成。现代国民教育体系基本完善，全民受教育程度和创新人才培养水平明显提高。人人享有基本医疗卫生服务，全民健康水平明显改善。城乡就业服务体系更加健全，就业比较充分；覆盖城乡的社会保障体系基本建立，人人享有基本生活保障。城乡居民生活水平和质量有较大提高。社会管理体系不断完善，社会更加和谐稳定。

　　三、２００８年的政府工作

　　２００８年，是新一届政府的开局之年，也是推进合肥又好又快发展的关键一年。我们要全面贯彻落实党的十七大精神，坚持以科学发展观为指导，坚持好字优先不动摇，坚持加快发展不动摇，坚持解放思想不动摇，坚持改革开放不动摇，变压力为动力，立足合肥自身优势，抢抓机遇，开拓进取，真抓实干，全力推动经济社会发展再上新台阶。

　　２００８年经济社会发展主要预期目标为：地区生产总值增长１５％，财政收入增长１５％，固定资产投资增长２５％，实际利用外资增长１４％，社会消费品零售总额增长１８％以上，城镇居民人均可支配收入和农民人均纯收入均增长１３％，城镇登记失业率控制在４．５％，人口出生率控制在１２‰以内，居民消费价格涨幅低于２００７年，单位ＧＤＰ能耗降低４％，化学需氧量、二氧化硫排放量达到省控指标。

　　实现上述目标，着重要抓好以下十个方面的工作：

　　（一）千方百计增加有效投入，促进经济又好又快发展

　　以培育优势产业、增强城市综合承载力、加强节能减排、民生工程和社会事业等为重点，坚持招商引资、重视民资、争取上资、扩大融资等多资并举，突出扩大有效投入，优化投资结构。一是抓住国家支持交通、能源、重大基础设施建设，鼓励科技创新、环境保护、节能减排等契机，加大项目对接力度，积极争取国家和省支持。二是拓展与各类金融机构的合作，开展多种形式的对接活动，积极争取信贷支持，扩大信贷投放。三是充分利用多层次资本市场，进一步加大拟上市企业辅导和扶持力度，推动优势企业上市融资，争取实现三至五家企业上市。四是整合各类招商资源，实现有效招商。抢抓国内外产业、资本转移和国家对中西部地区外经贸政策调整机遇，围绕重点领域、重点产业和重点载体，加大招商项目的策划、储备和推介力度，力争引进一批重大项目。切实做好招商服务基础工作，增强引资实效。五是加强政策引导和扶持力度，进一步激活和扩大民间投资，掀起全民创业热潮。六是进一步完善项目推进机制，落实项目建设责任制，实施重大项目调度制度，着力解决影响项目推进的突出问题，加快“１３４６”行动计划等项目建设。

　　（二）加快发展县域经济，推进新农村建设再上新台阶

　　坚持城乡统筹，加大对县域经济支持力度，鼓励城市企业、人才到县域发展。坚持以工业化为方向，以城市大工业为依托，以园区为载体，继续大力发展先进制造业、配套产业、食品及农副产品加工业。加快发展现代农业，促进农业产业结构进一步优化。一是大力发展现代种植业，进一步优化种植结构，着力提升蔬菜业，重点支持以“一村一品”、“一乡一业”为主的设施农业，新增设施栽培１．５万亩、露地菜３万亩；巩固林果业，新建苗木花卉生产基地２万亩，造林１．１５万亩。二是加快发展现代养殖业，加大乳业、生猪、家禽、龙虾、饲料、良种六大产业体系建设，组织实施“双百亿”工程，全面推进无规定动物疫病省级示范区建设。三是加快发展现代农副产品加工业，培育引进一批竞争力、带动力强的大型龙头企业，增强产业竞争力。四是加快开发现代休闲农业，依托山、水、园、林、庄等资源，挖掘农业生态观光潜力，提高农业综合效益。五是加快发展现代生态循环农业，大力开发以秸秆为主要原料的生物质肥料、饲料产业，发展农村沼气事业。六是加快建设现代农业服务体系，大力发展与现代农业相适应的经营主体，重点培育３０家市级农民专业合作经济组织和一批市级农民创业带头人；加强农村劳动力技能培训，继续实施“阳光工程”，提高转移就业率。

　　继续加大新农村建设投入高标准打造新农村建设示范点，推进示范点建设由中心村向基层村延伸。围绕改善农村居住条件和生态环境，加大农地开发整理和旧村庄整治力度，加快实施“村庄环境整治行动计划”。加强农田水利基本建设，重点建设高塘湖综合治理等防洪保安工程和５５座中小水库除险加固工程。基本实现“村村通水泥路”。加快城乡对接，继续大力推进城市水、气、热、污水管网等设施向县域城镇延伸。引导上派、店埠、北部组团呼应城市快速道路建设，加快城市副中心规划建设步伐。

　　（三）加速实施“工业立市”战略，促进工业经济快速发展

　　继续改进完善新型工业化发展的政策措施，鼓励加大工业投入，促进工业投资较快增长，突出重点，确保新开工一批、建成一批、发展一批。大力支持汽车、家电、装备制造等支柱产业加快技术改造，扩大产业规模，提高配套能力。积极建设国家级汽车及零部件出口基地城市，抓好江汽零配件产业园等一批重点项目建设。以申报中国家电产业基地为契机，加快家电产业集聚发展。坚持重点企业现场办公会制度，推动骨干企业加快发展，打造一批主业突出、实力雄厚、竞争力强的大企业大集团。完善中小企业服务体系，继续组织实施“小巨人”培育工程，选择一批具有一定基础、成长性较好的中小企业，开展“一企一项”的技术改造和技术创新。切实加强工业运行调度，继续抓好八大产业、３０户重点骨干企业、３０余种重要产品的监测分析，保障煤电油运等生产要素供给。围绕建设“千亿产值开发区”、“百亿产值工业园区”，加强开发园区统筹规划建设，进一步完善水、电、气、热、污水处理等基础设施和物流、金融、法律等公共服务平台，推进工业集中厂房建设，增强园区的产业承载力和综合服务能力。

　　（四）继续推进“大建设”，进一步提升城市综合承载力

　　围绕建设现代化滨湖大城市目标，统筹编制重大基础设施、生态环境、市场建设、产业布局等规划。继续加快区域综合交通枢纽建设，开工建设新机场、合肥港综合码头和合蚌高速铁路，建成合武高铁，完成合淮阜高速市区段。继续推进城市出入口道路建设，建成合六路，开工建设合店、合白、合水（合双段）路，完善“１４１”组团快速交通网。全面建成金寨路高架，加快建设包河大道、庐州大道、方兴大道、锦绣大道、紫云路。进一步完善主城区路网结构，全面提速在建项目，加快实施一环畅通工程，开工二环改造等一批道路工程。大力推进老城区改造，重点是加快危旧小区及城中村改造步伐，基本完成安纺一、三村改造，启动实施劳动村、老火车站区域等改造工程。继续大力推进四大组团及政务文化新区建设，进一步完善其基础设施配套和服务功能。加快滨湖新区中心医院、廉租房和经济适用房、世纪城、省农委、网通、广电、高速公路总公司大楼等项目建设，加快水、电、气、热等基础设施建设，尽快完善新区功能。积极推进“川气入肥”工程，加快建设六水厂，建成市府广场ＢＲＴ公交枢纽，开工建设杏花泵站排涝工程。

　　（五）加快科技创新型试点市建设，增强自主创新能力

　　认真贯彻落实部际协调小组会议精神，积极争取国家和省政策、资金、项目支持。深化产学研合作，健全院（校）地合作机制，围绕八大重点产业，建立１０个产学研战略联盟。充分发挥科技创新基金导向作用，继续实施创新型企业培育计划，推进电子信息、新材料、新能源等高新技术产业集群快速成长，使之成为产业发展主力军，全年新增高新技术企业６０家。强化科技创新服务体系建设，七大创新平台全面建成并发挥作用；积极引进国内外创新服务中介机构；完善损失补偿办法，建立风险投资机构和科技企业担保体系；认真办好第八届高新技术项目—资本对接会，积极争取高新区列入国家非上市中小企业股权挂牌交易试点；继续加快科技创新示范区基础设施建设，推进资金、项目、人才集聚，实施一批科技创新工程。积极创建国家知识产权示范城市，提升具有自主知识产权专利的数量和质量，建立国家专利技术交易展示中心。建成现代科技馆。

　　（六）深化重点领域改革，进一步增强发展活力

　　加大改革创新力度，积极申报国家创新型综合配套改革试点市。继续推进行政管理体制改革，把机关效能建设向纵深推进，全面整合政务类与事业类服务热线，不断优化政务环境。进一步推进国库集中支付等公共财政制度改革。坚持市、县（区）两级联动，深化投融资体制改革，壮大建投、国资等融资平台实力。继续加快发展招投标市场，理顺监管体制，着力建设区域性招投标平台。进一步完善国有资产监管体制，提升国有资本运行质量和水平。积极扶持非公有制经济发展，引导民营企业培育核心竞争力，走集约化生产、规模化经营之路。全面加快合钢集团存续企业、国风集团等大企业改革步伐，除公用事业外，其余国有企业年内全部完成改革任务。加快城市管理体制改革，明确划分市区两级建设管理权限，实行“事权下放、重心下移、属地管理”。继续分类推进事业单位改革，加快市政、园林、文化单位改革步伐。深化农村金融改革，加快组建三县农村合作银行，积极试办村镇银行，提升农村金融服务功能。

　　（七）推进环境综合治理，建设生态宜居型城市

　　转变经济发展方式，促进能源、资源节约集约利用。建立节能减排监测与责任体系。积极推进科研院所和高校与企业合作，以电力、钢铁等六大行业为重点，加快节能减排技术改造。继续开展循环经济试点工作。大力推广新型墙体材料及建筑节能产品，加快发展节能省地型住宅和公共建筑，实施城市垃圾发电项目。加大基本农田保护及闲置土地清理力度，层层落实依法管地用地的责任制。严格实行工业用地最低限价制度，提高土地投资强度和利用效率，推进土地资源的节约集约利用。

　　以巢湖综合治理为龙头，强化水环境治理，加大治污投入，实施巢湖沿岸生态治理工程。继续推进南淝河截污工程，开工建设四里河、板桥河、二十埠河、十五里河综合治理工程，实施西南部生态补水工程。加快建设十五里河污水处理厂一期、望塘污水处理厂二期工程，开工建设蔡田铺污水处理厂一期续建工程，实施城市污水处理厂除磷脱氮改造。加快主城区、开发园区及四大组团污水管网的建设与对接。继续深化饮用水源地专项整治工作。积极推广清洁生产，限期淘汰集中供热管网范围内的燃煤锅炉。加强重点污染源监管，严肃查处违法排污行为，坚决关闭高污染、高能耗“十五小”、“五小”企业。继续抓好城市油烟、噪声污染控制，加快公交、出租车“油改气”步伐。启动外环森林生态景观长廊建设，完成大蜀山森林公园建设，高标准实施精品道路绿化工程，续建高压走廊绿化项目，新建园林绿地一万亩，栽植乔木１００万株。

　　（八）改造提升传统商贸服务业，大力发展现代服务业

　　编制实施中长期商业发展规划和新一轮商业网点布局规划，加快城乡市场体系建设。着力建设一批高档次、综合性大型商业设施，继续加大专业特色市场建设力度。改造升级长江中路沿线等老城区商业设施，加快建设滨湖新区、政务文化新区等新城区商业网点，推进社区商业“双进工程”和农贸市场改造工程。继续实施“万村千乡”、“新网”工程，形成城乡流通网络新格局。加快发展现代物流等生产性服务业，重点扶持十个专业物流基地和园区。加快金融业发展，力争引进一至两家外资银行。继续推进全省旅游中心城市建设，加快打造旅游集散地和旅游目的地推动环巢湖旅游开发，加快三河等景区规划建设；深度开发科教游、文化游、工业游，形成一批乡村特色旅游品牌。提升会展业的国际化水平，培育和壮大会展品牌，力争新举办一至两个具有较大影响的全国性会展。

　　（九）积极推进精神文明建设，协调发展各项社会事业

　　以提高全社会文明程度为目标，深入开展群众性精神文明创建和学习型城市创建活动，大力倡导社会公德、职业道德和家庭美德，以社会主义荣辱观引领时尚，切实把社会主义核心价值体系融入国民教育和精神文明建设全过程。继续推进市容、交通、农贸市场等综合整治，力争在省会城市率先进入全国文明城市行列。

　　高度重视文化建设。积极推进文化艺术中心、广电中心、渡江战役纪念馆、少儿活动中心等设施建设，全面启动农村公共文化服务体系建设。制定文化产业发展政策，加快发展休闲、娱乐、文博业，积极引进高水平演艺团队，着力打造一批精品文化娱乐项目。继续实施城区中小学布点规划，进一步完善农村中小学布点调整工作，加快资源整合，促进均衡发展。大力推进滨湖三校二期和合肥八中新校一期工程建设，确保秋季建成使用。继续大力支持合肥学院的建设和发展。更加关注农民工子女、留守儿童教育，关心支持幼儿教育、特殊教育和民族教育。大力推进职教基地规划建设，加快基础设施建设和院校入驻进度。依法管理各类民办教育，支持引导民办教育健康发展。全面落实卫生专项规划，启动健康城市建设。大力推进国家社区卫生服务体系建设重点联系市工作，进一步提高新型农村合作医疗制度的保障水平和服务能力。继续推进市职业病防治医院等卫生基础设施建设。广泛开展全民健身运动，精心组织奥运火炬合肥传递活动，办好市第九届体育运动会。建立健全流动人口计生管理新机制，继续推进全国婚育新风进万家示范市建设。做好全民国防教育、民兵预备役、国防动员和人防工作，巩固双拥模范城创建成果。认真贯彻落实党的民族宗教政策，促进民族团结，依法加强宗教事务管理。做好全国第二次经济普查任务准备工作，完成《合肥市志》续编。积极推动外事、侨务、对台事务、防震减灾、气象、档案、保密、妇女儿童、未成年人保护、老龄、残疾人等事业发展。

　　（十）着力保障和改善民生，促进和谐社会建设

　　深入贯彻《就业促进法》和《劳动合同法》，大力促进下岗失业人员、高校毕业生、被征地农民等群体就业再就业，全年新增城镇就业岗位七万个；继续创建“充分就业社区”，及时消除“零就业家庭”；积极实施“劳动合同三年行动计划”，切实维护劳动者合法权益。

　　大力实施各项民生工程。做好城镇职工医保、城镇居民医保和新型农村合作医疗的制度衔接，扩大非公经济组织、城镇灵活就业人员和农民工参保面，实现城镇居民医疗保险全覆盖。完善被征地农民基本生活保障制度和农村社会养老保险制度。巩固扩大退管示范社区创建成果，进一步提高企业退休人员社会化管理服务质量。完善城乡社会救助体系，加强城市低保动态管理，健全农村低保制度；加快实施农村五保供养“５１５”工程，提高集中供养率；实行高校和中职学校贫困学生资助及贫困白内障患者复明工程；完善流浪乞讨人员救助管理机制。继续开展“慈善一日捐”活动，推动慈善事业发展。强化住房保障制度，进一步扩大覆盖面，基本完成城区大建设拆迁安置房建设。进一步完善粮食、生猪等储备体系，大力实施“菜篮子”工程，增加有效供给，稳定市场物价。继续实施农村安全饮水工程，解决２０．８万人口安全饮水问题。大力推进“村村通班车”工程，切实改善农村出行环境，促进农村物流畅通。

　　进一步加强科技强警示范城市和公安“三基”工程建设，完善社会治安防控体系，切实保障人民群众生命财产安全。进一步加强社会建设和管理，重点抓好流动人口、出租房服务管理工作。全面开展矛盾纠纷排查化解、党政领导干部公开接访工作，解决群众合理诉求。建立涉及群众利益的重大事项社会稳定风险评估机制，努力从源头上减少矛盾发生。继续加强产品质量和食品药品安全工作，整顿和规范市场经济秩序。强化安全生产责任，加强重大危险源监控，建立隐患排查治理长效机制，严防重特大事故发生。深入推进和谐社区建设，积极创建全国和谐社区建设示范市；扩大农村社区建设试点，积极开展村务公开和民主管理示范单位创建活动。

　　四、建设勤政、务实、高效、廉洁的服务型政府

　　坚持依法行政、高效行政，着力转变职能，理顺关系，提高效能，逐步形成权责一致、分工合理、决策科学、执行顺畅、监督有力的行政管理体制，不断提高政府管理经济社会事务的能力和水平。

　　一要解放思想，勇于创新。始终坚持把解放思想放在首位，立足于形势的变化，立足于经济社会发展的实际，坚决清除一切妨碍发展的思想障碍，摒弃一切束缚发展的做法和规定，革除一切影响发展的体制和弊端，牢固树立科学发展、和谐发展的新思想、新观念，以思想的不断解放促进经济的发展和民生的改善，以观念的不断更新激活体制的潜力与机制的活力。大力营造和保护创新的环境和氛围，使崇尚创新、追求创新成为广大干群特别是各级领导干部的自觉行动，以蓬勃向上的朝气、敢闯敢试的锐气、开拓进取的勇气做好各项工作。

　　二要深化改革，提高效能。全面履行好政府职责，把抓经济的主要精力放到创造良好发展环境上来，把政府工作的重点、政策支持的重点、财力保障的重点，向公共安全、社会事业、生态建设、就业保障等方面倾斜。合理划分市与县（区）的事权和财权，激发基层活力。推进政府机构改革，探索实行“大部门”体制，着力解决部门之间职责交叉、权责脱节和政出多门的问题。规范行政审批及行政处罚自由裁量权，积极构建行为规范、流程便捷、监督有力的审批新机制，努力降低行政成本。继续优化各级政府和部门的运行方式及工作流程，严格落实服务承诺制、首问责任制等制度。严格控制各类检查评比，下大力气压缩会议、减少文件。加强各级政府门户网站建设，拓宽网上审批范围，提高在线办事水平和能力。

　　三要依法行政，强化责任。坚持科学决策、民主决策，健全重大决策事项专家咨询制度、听证制度和公示制度，提高决策透明度和公众参与度。严格执行《合肥市人民政府行政问责暂行办法》，落实行政问责制度。坚持和完善民主法制建设，自觉接受人大及其常委会的依法监督，重视和支持人民政协发挥政治协商、民主监督和参政议政作用，自觉接受新闻媒体和人民群众监督，加强与各民主党派、工商联和无党派人士的联系，充分发挥工会、共青团、妇联等人民团体的桥梁和纽带作用。全面推进依法行政，认真贯彻实施《合肥市（２００８－２０１２年）推进依法行政五年规划》，进一步提高公务员的法制观念和依法办事能力。加强行政执法部门建设，完善综合执法，加大相对集中行政处罚权工作。认真贯彻国务院《信息公开条例》，全面推进政务公开。

　　四要廉洁奉公，执政为民。坚持标本兼治、综合治理、重在治本、重在建设的廉政工作要求，加强权力制约和监督，真正做到用制度管权、用制度管事、用制度管人。发挥行政监察职能，查处违纪违法案件，加大纠风工作力度，着力解决环境保护、土地征用、房屋拆迁、企业改制等方面损害群众利益的问题。加强审计工作，深化预算执行审计，完善经济责任追究制度。深入贯彻实施《公务员法》，强化公务员队伍建设，积极探索并形成跨部门公务员交流机制。牢固树立公仆意识，坚持以人民利益为重，以合肥发展为上，凝心聚力不分神，埋头苦干不浮躁，真正把服务人民的宗旨落实在行动中，把“三大推进”的要求体现到实践中，切实肩负起执政为民、执政兴市的历史使命。

　　各位代表，多年来，全市人民精诚团结，克服了重重困难，创造了一个又一个辉煌业绩，展现出昂扬向上的精神风貌，我们这座城市从来没有像今天这样充满生机与活力。未来五年的宏图已经绘就。肩负时代的使命和人民的重托，我们深感事业的光荣和责任的重大。让我们更加紧密地团结在以胡锦涛同志为总书记的党中央周围，在省委、省政府和市委的坚强领导下，开拓创新，扎实工作，在新的征途上，创造新的业绩，谱写出无愧于时代要求、无愧于人民期望的崭新篇章！
